# NF-κB signalling and cell fate decisions in response to a short pulse of tumour necrosis factor

**DOI:** 10.1038/srep39519

**Published:** 2016-12-22

**Authors:** Robin E. C. Lee, Mohammad A. Qasaimeh, Xianfang Xia, David Juncker, Suzanne Gaudet

**Affiliations:** 1 Department of Cancer Biology and Center for Cancer Systems Biology, Dana Farber Cancer Institute, Boston, MA 02215, USA; 2 Department of Genetics, Harvard Medical School, Boston, MA 02115, USA; 3 Biomedical Engineering Department, McGill University, Montréal, Québec, H3A 2B4 Canada; 4 McGill University and Genome Québec Innovation Centre, Montréal, Québec, H3A 0G1, Canada; 5 Department of Neurology and Neurosurgery, McGill University, Montréal, Québec, H3A 2B4 Canada

## Abstract

In tissues and tumours, cell behaviours are regulated by multiple time-varying signals. While in the laboratory cells are often exposed to a stimulus for the duration of the experiment, *in vivo* exposures may be much shorter. In this study, we monitored NF-κB and caspase signalling in human cancer cells treated with a short pulse of Tumour Necrosis Factor (TNF). TNF is an inflammatory cytokine that can induce both the pro-survival NF-κB-driven gene transcription pathway and the pro-apoptotic caspase pathway. We find that a few seconds of exposure to TNF is sufficient to activate the NF-κB pathway in HeLa cells and induce apoptotic cell death in both HeLa and Kym-1 cells. Strikingly, a 1-min pulse of TNF can be more effective at killing than a 1-hour pulse, indicating that in addition to TNF concentration, duration of exposure also coordinates cell fate decisions.

Although many experiments characterize cellular responses to continuous exposure to a stimulus, stimuli in normal tissues and tumours are often time-varying. One such stimulus is Tumour necrosis factor (TNF). *In vivo*, TNF released after lipopolysaccharide (LPS) exposure is rapidly cleared, and the half-life of injected TNF in the blood is a few minutes^1^,^2^,^3^,^4^. TNF is a pro-inflammatory cytokine that was originally identified as an anti-tumour agent because of its cell-killing activity^5^,^6^,^7^,^8^,^9^,^10^,^11^. Despite some anti-tumour properties, TNF-cancer connections are complex as TNF-associated inflammation is also linked with progression of certain cancers (e.g. ref. 12; reviewed in refs 13 and 14). Likewise, while TNF is important for normal immune cell functions^5^, chronically elevated TNF is associated with autoimmune diseases, many of which are treated with anti-TNF therapies^15^.

TNF regulates diverse cellular behaviours such as migration, differentiation and apoptosis^5^,^6^,^7^,^8^. This diversity of responses may stem from the fact that TNF initiates a paradoxical network of pro-survival and pro-apoptotic intracellular signals. At the plasma membrane, the TNF-initiated “Complex I” induces nuclear translocation of canonical NF-κB dimers^16^,^17^ to drive transcription of many inflammatory and pro-survival genes^18^. Following TNFR1 internalization, cytoplasmic “Complex II” initiates the extrinsic apoptosis pathway by recruiting and activating initiator caspases^17^,^19^,^20^. Little is known about how the duration of exposure to TNF affects the balance between NF-κB and caspase signalling.

Experiments in mouse embryonic fibroblasts have shown that NF-κB activation long outlasts treatment with a 1-, 2- or 5-min pulse of TNF and that, compared to continuous treatment, NF-κB activation is nearly indistinguishable in amplitude or timing when quantified by electromobility shift assays^21^. Nevertheless, the NF-κB-driven transcription profile of neuroblastoma cells is sensitive to TNF treatment duration – a 5-min TNF pulse induces transcription of early, but not late, response genes^22^. Building an impressive dataset, Kellogg and colleagues reported that transient exposure of mouse embryonic fibroblasts to TNF or LPS may follow an ‘area-rule’ for NF-κB nuclear translocation whereby the fraction of responding cells is proportional to the product of stimulus concentration and duration^23^. Yet, the strength of NF-κB activity in single cells treated with a pulse of TNF is not well-characterized experimentally and it is not known whether persistent vs. transient TNF exposure influences cell fate.

Here, we set out to determine the TNF pulse duration required for NF-κB activation in single human cancer cells, and study how pulse duration affects TNF-induced apoptosis. Using single-cell data, we quantified the threshold of NF-κB nuclear translocation required for inducing early gene transcription. We show that for a high TNF concentration, a 10-sec pulse was sufficient for NF-κB activation but a longer pulse is required at a lower TNF concentration. Extrinsic apoptosis was also strongly activated by a short TNF pulse. In HeLa cells, a 1-min TNF pulse induced apoptosis with a potency similar to that of a 10-hr treatment whereas a 30- or 60-min pulse was less effective in cell killing. For Kym-1 cells, a 30-sec pulse of TNF induced as much death as continuous stimulation. Our study reveals that the duration of TNF exposure influences the TNF-induced cell fate decision.

## Results

### A brief TNF pulse induces nuclear localization of RelA

**Figure 1:**
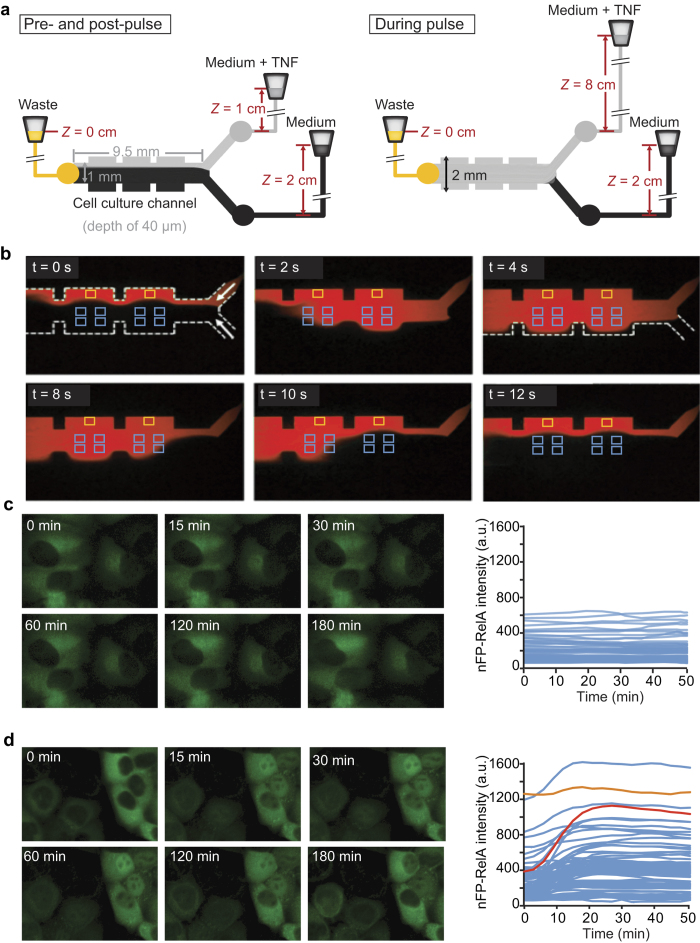
A microfluidic system to study cellular responses to a short pulse of TNF. (**a**) Schematic of the Y-junction microfluidic chip; the chambers are 2 × 2 mm^2^ separated by a 1 mm wide connection. The microfluidic channels and chambers have a 40-μm depth. The flow is gravity-driven and in the default operating mode, before and after the pulse of TNF, the ‘Medium’ reservoir is positioned higher than ‘Medium + TNF’ (2 cm vs. 1 cm, left). The ‘Medium + TNF’ reservoir is manually raised to 8 cm for the duration of the pulse (right), and then lowered again. (**b**) Time-lapse epifluorescence images of the ‘Medium’ and ‘Medium + TNF’ laminar streams during a pulse of TNF; the ‘Medium + TNF’ stream was visualized using Alexa647-conjugated BSA (red; see Supplementary Movie S1). Boxes mark examples of regions of the channel that would be imaged to track cells continuously exposed to TNF (yellow) or cells exposed to a pulse of TNF (blue). (**c** and **d**) Time-lapse images of EGFP-RelA HeLa cells treated with a 1-min pulse of Alexa647-BSA only, or a 30-sec pulse of Alexa647-BSA and 100 ng/ml TNF (left panels of c & d respectively). The time course of mean nuclear EGFP-RelA intensity (nFP-RelA intensity) for each cell was quantified from time-lapse images (right panels; n = 67 cells in (**c**), n = 82 cells in (**d**) from one of four imaging positions from one representative experiment). The nEGFP-RelA time courses for two cells with different dynamics are highlighted in red and orange; a.u., arbitrary units.

We designed and built a simple microfluidic system that uses laminar flow^24^ to provide spatiotemporal control over TNF delivery in cell cultures. Our device consists of two inlets for culture medium with and without TNF, a three-chamber cell culture channel, and a single outlet (Fig. 1a and Supplementary Fig. S1a). Hydrostatic pressure differences between inlet reservoirs in the default operating mode caused most of the channel to be exposed to ‘Medium’ (Fig. 1a,b, t = 0 sec), with a narrow band of positive-control cells continuously exposed to a laminar stream of ‘Medium + TNF’ (Fig. 1b, yellow boxes). To expose the rest of the cells to a TNF pulse, the ‘Medium + TNF’ reservoir was temporarily raised, increasing its hydrostatic pressure (Fig. 1a, right, Fig. 1b, blue boxes). This reservoir also contained Alexa647-conjugated BSA allowing us to track the TNF-containing stream by imaging (Fig. 1b and Supplementary Movie S1). With this simple system, we reproducibly achieved TNF pulses as short as 10 sec.

To monitor TNF-induced NF-κB activation, we seeded the device with EGFP-RelA-expressing HeLa cells (Supplementary Fig. S1c) and quantified nuclear EGFP-RelA from time-lapse images as described previously^25^. The calculated flow shear forces in the device were small (<0.2 Pa for imaged areas; Fig. 1b, yellow and blue boxes and Supplementary Fig. S1b), and therefore unlikely to stimulate the NF-κB pathway via mechanotransduction. Indeed, a mock 1-min pulse of medium without TNF had no observable effect on EGFP-RelA nuclear abundance (Fig. 1c). In contrast, a 30-sec pulse of 100 ng/ml TNF resulted in transient and variable EGFP-RelA nuclear translocation in most cells (Fig. 1d and Supplementary Movie S2). Single-cell nuclear RelA time courses resembled those observed by us and others in response to continuous or 5-min TNF treatments^22^,^25^,^26^,^27^,^28^,^29^, showing that a very short TNF pulse induces NF-κB nuclear translocation in HeLa cells and demonstrating that we could monitor it in our device.

### Defining a transcription-inducing EGFP-RelA translocation

**Figure 2:**
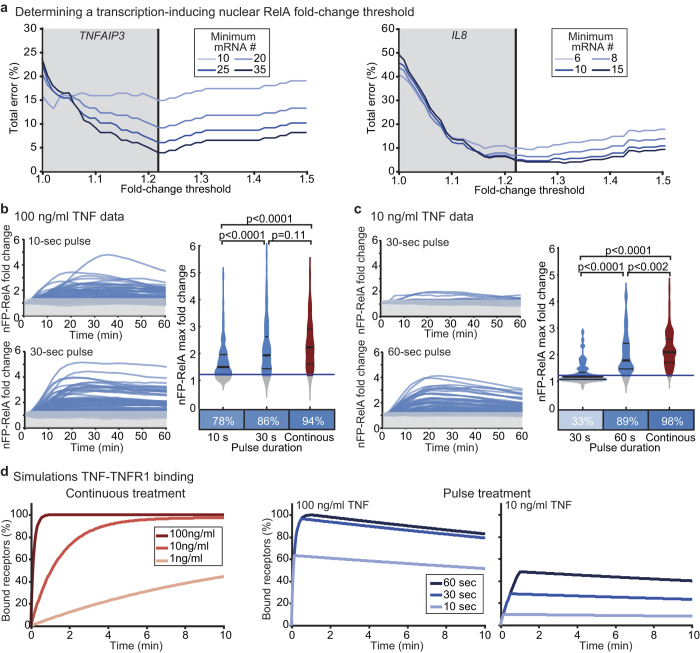
The duration of TNF treatment required to elicit a transcriptionally significant NF-κB response is short but concentration-dependent. (**a**) Total error in determining whether a cell underwent a transcription-inducing response to TNF based on same-cell EGFP-RelA translocation and transcripts number data for *TNFAIP3* (left) and *IL8* (right) (data from ref. 25). The error was evaluated for several mRNA number cut-offs (distinguishing responsive vs non-responsive; shades of blue), as a function of the fold-change threshold (x-axis). Final total error estimates used cut-offs of 35 (*TNFAIP3*) and 8 (*IL8*) mRNAs, which appropriately separated distributions from untreated vs. TNF-treated cells (Supplementary Fig. S2). Grey zone indicates fold-change levels deemed ‘non-responsive’, below the transcription-inducing threshold determined by minimizing total error (vertical black line); data collated from three independent experiments totalling n = 192 cells (*TNFAIP3*) and n = 203 cells (*IL8*). (**b** and **c**) Single-cell nuclear EGFP-RelA (nEGFP-RelA) time courses after a 100 ng/ml (**b**) or 10 ng/ml (**c**) TNF pulse with indicated duration (n = 67 and n = 82 cells from one of four imaging frames in one experiment from at least two independent replicates). Gray zones mark regions below the 1.22-fold cut-off (‘non-responsive’). Violin plots show relative frequency distributions of nuclear EGFP-RelA fold-change in ‘responsive’ (blue, TNF pulse; red, continuous exposure) or non-responsive cells (grey). Median (thick black line) and upper and lower quartiles (thin black lines) are marked. Percentages of ‘responsive’ cells are shown in heatmaps below; p-values are for a Kolmogorov-Smirnov test of the equality of each distributions pair. Each condition combines data from at least two independent replicate experiments, with between 69 and 389 total cells; violin plots were generated in MatLab from smoothened histograms (normal kernel). (**d**) Time courses of fraction of bound receptors in simulations of HeLa cells with continuous TNF treatment (1, 10 or 100 ng/ml TNF, light, medium and dark red respectively; left). Time courses of the fraction of bound receptors in simulations of HeLa cells exposed to a TNF pulse at 100 ng/ml (middle) or 10 ng/ml (right) with indicated duration (10, 30 and 60 sec, light, medium and dark blue respectively).

Cell-to-cell variability of nuclear EGFP-RelA following a 30-sec TNF pulse was substantial. Some cells had large changes in nuclear EGFP-RelA abundance (Fig. 1d, red trace) while others had high initial nuclear EGFP-RelA and only a small TNF-induced increase (Fig. 1d, orange trace). We set out to determine which cells were truly ‘responsive’ to the TNF pulse, cells in which NF-κB activation should result in target gene transcription. We leveraged our published dataset of same-cell EGFP-RelA nuclear translocation time courses and target transcript numbers^25^ to establish the EGFP-RelA nuclear translocation threshold under which a cell is unlikely to induce NF-κB-dependent gene transcription. In that study, we determined that the maximal fold change of nuclear RelA (ratio of maximal to initial nuclear RelA in a cell) is predictive of transcript number for TNF-driven and RelA-dependent early response genes^25^. We therefore evaluated the error rate in determining whether or not a cell had a transcription-inducing EGFP-RelA translocation (‘responsive’ vs. ‘non-responsive’) while varying two parameters: fold-change threshold and cut-off value for transcript number. Although our earlier data were from cells treated continuously with TNF, we reasoned that if a given fold change is unlikely to induce transcription under continuous exposure, then it is also unlikely to induce transcription after a short pulse. We found a minimum of total error at ~1.22-fold-change for both *IL8* and *TNFAIP3*, two NF-κB-inducible genes with no or few transcripts in unstimulated cells (Fig. 2a and Supplementary Fig. S2; ~5% error). This fold-change threshold showed little dependence on the transcript number cut-off value, although values of 8 and 35 transcripts discriminated well between untreated and TNF-treated distributions for *IL8* and *TNFAIP3* respectively (Fig. 2a, and Supplementary Fig. S2a,b). Applying this threshold, we determined that the red-trace cell in Fig. 1d was responsive to the 30-sec TNF pulse (max fold change = 2.94), while the orange-trace cell was non-responsive (max fold change = 1.06) despite having overall greater nuclear EGFP-RelA abundance. Altogether, our analysis shows that the NF-κB system can sense and respond to an increase of nuclear RelA of as little as ~20%.

### The duration of TNF exposure required for NF-κB activation is concentration-dependent

We next assessed EGFP-RelA nuclear translocation in response to different TNF pulse durations (Fig. 2b,c). We found that 78% and 86% of cells were responsive when exposed to 100 ng/ml TNF for 10-sec or 30-sec respectively, versus 94% for continuous treatment. Importantly, fold-change distributions for a 30-sec pulse or continuous treatment with 100 ng/ml TNF were not significantly different (Fig. 2b). At 10 ng/ml TNF, cells required a longer pulse for transcription-inducing EGFP-RelA translocation; only 33% of cells were responsive after a 30-sec pulse (Fig. 2c). This percentage climbed to 89% for a 60-sec pulse and to 98% for continuously treated cells yet in this scenario, the nuclear EGFP-RelA fold-change distributions were still significantly different.

In contrast to response distributions for a short TNF pulse, distributions of nuclear EGFP-RelA fold change observed under continuous treatment with 10 ng⁄ml vs. 100 ng⁄ml TNF are similar (Fig. 2b,c; p = 0.30, unpaired Kolmogorov-Smirnov test). However, others have reported that TNF concentration impacts both the fraction of cells responding as well as certain response parameters such as the lag before RelA nuclear entry^28^,^30^. Applying our threshold for transcription-inducing EGFP-RelA translocation, we found that when we reduced the TNF concentration to 1 or 0.1 ng/ml fewer cells responded (Supplementary Fig. S3a). Moreover, the average nuclear EGFP-RelA fold change in responding cells decreased with TNF concentration (Supplementary Fig. S3b). This suggests that for continuously exposed cells, TNF concentration is reflected both in the percentage of responding cells and in the NF-κB pathway response amplitude, although this response saturates by 10 ng⁄ml TNF.

To understand how ligand-receptor interactions vary with TNF concentration and pulse duration, we simulated TNF-TNFR1 reversible binding kinetics as a general bimolecular surface reaction^31^. Simulations using previously determined binding constants for ^125^I-labeled TNF with TNFR1 in HeLa cells^32^ showed that receptors could be saturated within 30–60 sec with 100 ng/ml TNF, but only after 5–8 min with 10 ng/ml TNF (Fig. 2d). Only ~50% of receptors may be bound after a 60-sec pulse at 10 ng/ml TNF (Fig. 2d), explaining the observed concentration-dependence of the minimal TNF pulse duration for NF-κB activation. By running similar simulations over a range of receptor numbers (for both TNFR1 which is nearly ubiquitously expressed and TNFR2, which is more restricted in expression and absent in HeLa cells^32^) as well as a range of parameter values, we found that this result is not sensitive to receptor number and is robust to large variation in parameter values (Supplementary Figs S4 and S5). Notably, in all contexts the dissociation of TNF from TNFR1 is slow, suggesting that complexes should persist long after a pulse (Fig. 2d). Because internalization of TNF-bound receptors begins as early as several minutes after exposure to TNF^17^,^33^, our simulations predicted that a short TNF pulse could be sufficient to activate later signalling events such as the assembly of pro-apoptotic Complex II.

### Apoptosis occurs in response to a short pulse of TNF

**Figure 3:**
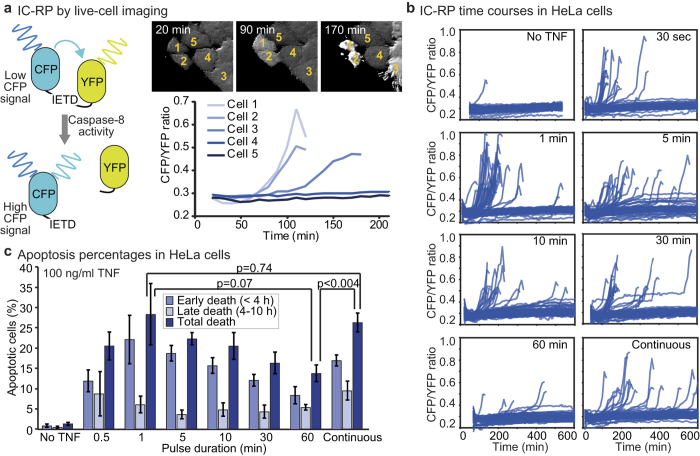
A short pulse of TNF induces significant caspase activity and cell death in HeLa cells pre-treated with IFNγ. (**a**) Schematic diagram of the FRET-based reporter of initiator caspase activity (IC-RP; left^55^) as well as time-lapse images of five IC-RP expressing HeLa cells treated continuously with 100 ng/ml TNF after a 24-hr pre-treatment with 200 U/ml IFNγ (top right). Single-cell time courses of caspase activity are quantified by the CFP/YFP ratio (bottom right). (**b**) Single-cell time courses of caspase activity for HeLa cells exposed to a 100 ng/ml TNF pulse of indicated duration after a 24-hr pre-treatment with 200 U/ml IFNγ (n = 67 to 93 cells in each condition, data from one of five independent replicate experiments). (**c**) Bar graph of the average percentages of HeLa cells that die within the indicated time period after a 100 ng/ml TNF pulse of indicated duration which followed a 24-hr, 200 U/ml IFNγ pre-treatment. Error bars designate the S.E.M. for five independent replicate experiments, with cell numbers between 50 and 375 in each condition for each experiment. P-values are reported indicating that percentages of total cell death are not significantly different between 1-min pulse and continuous treatment (p = 0.80; paired two-tailed t-test) and that the percentage of cell death after a 60-min pulse is significantly lower than during continuous treatment (p < 0.004; paired one-tailed t-test) but not significantly lower than after a 1-min pulse (p = 0.07; paired one-tailed t-test).

To monitor caspase activity in single TNF-treated cells, we imaged HeLa cells expressing a FRET-based initiator caspase reporter (IC-RP^34^; Fig. 3a) and quantified IC-RP cleavage by the CFP/YFP ratio (Fig. 3a and Supplementary Movie S3). Cells were pre-treated with interferon-γ (IFNγ) then TNF-treated. IFNγ is a cytokine that sensitizes many cancer cell lines, including HeLa cells, to TNF-induced apoptosis, partly via increased initiator caspase-8 expression^35^,^36^,^37^,^38^,^39^ and, *in vivo*, TNF and IFNγ often co-occur as IFNγ serves to activate macrophages which are a major source of TNF^40^. To limit the effects of dynamic signals activated by the immediate response to IFNγ and thus isolate the role of TNF, cells were pre-treated with IFNγ for 24 hrs before TNF addition. IC-RP cleavage accumulation varied from cell to cell, likely, at least in part, because of natural variation in the abundance of apoptotic signalling molecules^41^,^42^,^43^,^44^. For cells that underwent TNF-induced apoptosis, cleaved IC-RP accumulated sharply and the CFP/YFP peak corresponded with apoptotic morphology before cell detachment, revealing the ‘time-of-death’. In cells that survived, CFP/YFP fluctuations were small (Fig. 3a). In continuous TNF treatment, we observed significantly more apoptosis at 100 ng/ml than at 10 ng/ml (Supplementary Fig. S6a) and therefore focused on 100 ng/ml TNF for our analysis of how the extent and timing of cell death responses vary with pulse duration.

Because TNF-induced apoptosis occurs over many hours, we increased experimental throughput by performing wash-out experiments in 96-well plates instead of performing them under flow condition in the low-throughput microfluidic system. The shortest pulse we could reproducibly impose was 30-sec and thus we quantified dynamics of IC-RP cleavage after a TNF pulse ranging from 30-sec to 60-min and for cells treated continuously. Consistently, we observed that a 1-min pulse was at least as effective at killing cells as 10-hr continuous TNF treatment (Fig. 3b,c). By contrast, less cell death was observed after a 60-min pulse of TNF (p < 0.004 vs. continuous treatment, paired one-tailed t-test), showing that shortening exposure to high-concentration TNF does not necessarily decrease its pro-apoptotic effect. In addition, cells that died in response to a TNF pulse of 30 min or less died earlier on average than continuously treated cells (Fig. 3c and Supplementary Fig. S6b).

**Figure 4:**
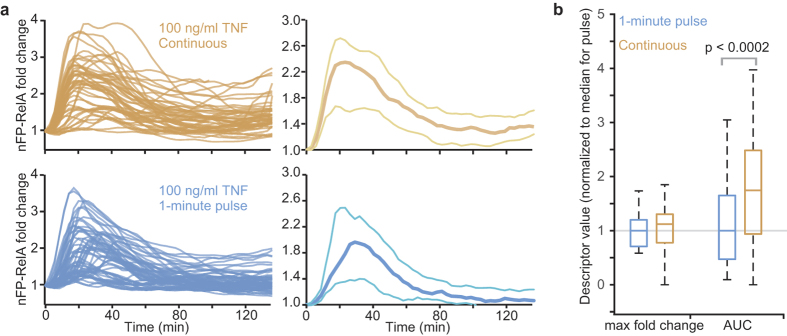
Duration of EGFP-RelA nuclear residence depends on duration of TNF exposure. (**a**) Single-cell nuclear EGFP-RelA time courses after continuous treatment (orange, n = 46 cells) or 1-min pulse (blue, n = 55 cells) of TNF (left). EFGP-RelA HeLa cells were treated only with TNF, see Figure S7 for time courses of nuclear EGFP-RelA from cells pre-treated with IFNγ. Average (thick line) and standard deviation (thin lines) of time courses plotted on the right. (**b**) Box plot of the distributions of the max fold change and area under the fold-change curve (AUC) for trajectories in (**a**) showing the median and top and bottom quartiles (box) as well as 5^th^ and 95^th^ percentiles (whiskers). P-value indicates that there is a significant difference in AUC between continuous- and pulse-treated cells (one-tailed t-test).

For cells to die earlier in response to a short exposure to TNF, TNF-induced signalling pathways are likely to be distinctly coordinated in at least a subpopulation of cells. Indeed, we found subtle but statistically significant differences in the short-term EGFP-RelA dynamics (as quantified by the max fold change in nuclear EGFP-RelA), as well as in the endogenous RelA distributions, for IFNγ-pre-treated cells exposed to a 1-min TNF pulse versus cells exposed to TNF continuously (Supplementary Fig. S7a,b). This is in contrast to what we observed in the absence of IFNγ pre-treatment where there was no statistically significant difference between a 1-min TNF pulse or continuous treatment, as expected based on our results for a shorter TNF pulse in the microfluidic device (Fig. 4 and Fig. 2b). Despite similarity in the max fold change in nuclear EGFP-RelA, examination of nuclear EGFP-RelA at later time points suggested that EGFP-RelA resides in the nucleus for a longer period under continuous TNF treatment (Fig. 4a and Supplementary Fig. S7c). We therefore compared the time-integrated nuclear EGFP-RelA signal, calculated as the area under the fold change of nuclear EGFP-RelA time course (AUC), to assess whether there are differences in longer term RelA dynamics. We found that both in the absence and presence of IFNγ-pre-treatment, continuous treatment with TNF led to prolonged residence of EGFP-RelA in the nucleus and thus a greater integrated signal (Fig. 4 and Supplementary Fig. S7c,d). Although it is likely that additional distinctions also lie elsewhere in the TNF-induced signalling network, it is possible that the shorter activation of RelA in the context of a response to a short pulse of TNF leads to a reduced pro-survival response and accelerated cell death.

### Efficient induction of apoptosis in response to a short pulse of TNF is concentration-dependent

**Figure 5:**
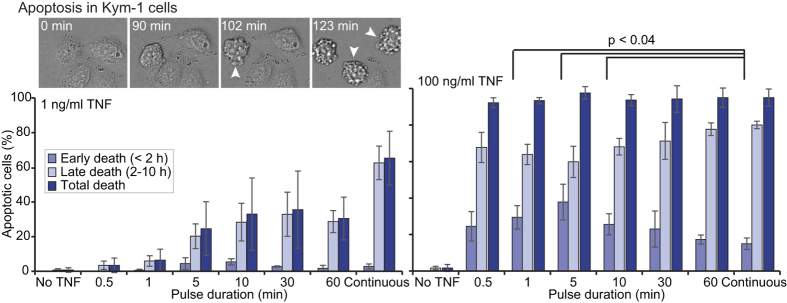
The sensitivity to a short pulse of TNF is dose-dependent in Kym-1 human rhabomyosarcoma cells. Time-lapse images of four Kym-1 cells treated continuously with 1 ng/ml TNF (top left). Time of cell death was noted as the first frame in which the cell took on a rounded morphology with membrane blebbing (arrowheads). Bar graphs of the average percentages of IFNγ-pre-treated Kym-1 cells that die within the indicated time period after a pulse of indicated duration with 1 ng/ml TNF (left) or 100 ng/ml TNF (right). Error bars designate the S.E.M. for n = 3 (1 ng/ml) or n = 4 (100 ng/ml) independent replicate experiments (between 25 and 100 cells were tracked in each condition for each experiment). All of the 1 ng/ml pulse durations, except the 30-min pulse (p = 0.07), induced significantly less cell death than continuous treatment (p < 0.015; paired one-tailed t-tests). For 100 ng/ml TNF, all of the pulse treatments induced as much cell death as continuous treatment (p > 0.25 for all; paired two-tailed t-tests) but a pulse of 1 min, 5 min or 10 min results in significantly more cell death within the first 2 hours after TNF treatment than continuous treatment (p < 0.04; paired one-tailed t-tests).

Our simulations of TNF-TNFR1 binding suggest that at low TNF concentrations much longer exposures might be required to achieve binding of TNF to a large fraction of TNFR1 (Fig. 2d). Therefore, we sought to test whether a cell line sensitive to low TNF concentrations, the Kym-1 human rhabdomyosarcoma cell line, would exhibit duration-dependent cell death at a low TNF concentration. Indeed, we found that at 1 ng/ml TNF, the fraction of Kym-1 cells that die increased with exposure duration (Fig. 5, left panel). However, at 100 ng/ml TNF, a 30-sec exposure to TNF induced as much cell death as continuous treatment (Fig. 5, right panel) and, as for HeLa cells, our Kym-1 data suggests that a short pulse of high-concentration TNF induces significantly earlier cell death than continuous treatment (Fig. 5 and Supplementary Fig. S8).

Taken together, our data show that a short pulse of high-concentration TNF is sufficient to induce substantial cell death in both HeLa and Kym-1 cells. The duration of this pulse in turn affects the efficacy and timing of the pro-apoptotic effect of TNF – cells treated with a brief pulse tend to die earlier than cells treated continuously. Although the extent of TNF-induced cell death after a 1-min pulse is comparable with continuous treatment, longer pulse durations are less effective in HeLa cells suggesting that the duration of cytokine exposure is an important mediator of TNF-induced signalling and cell fate.

## Discussion

In a tissue, cell may experience transient exposure to inflammatory cytokines as these are released in bursts by migrating tissue-resident immune cells and can be rapidly cleared. Using a simple microfluidic system, we characterized the cellular responses to different durations of exposure to TNF in single-cells. Our results demonstrate that the minimum pulse required for TNF-induced signal transduction is concentration-dependent, and that at high TNF concentrations, a pulse as short as 10–30 sec can induce significant NF-κB translocation and a pulse of 30 sec or 1 min can induce caspase activation and apoptosis. A similar concentration-dependence was recently reported for the treatment duration required for LPS-induced NF-κB activation in mouse embryonic fibroblasts and PDGF-induced PDGFR phosphorylation in NIH-3T3^23^,^45^ suggesting that this may generalize to other cell types and extracellular ligand systems.

We note here that although the maximal TNF concentration used in our study exceeds serum concentrations from patient samples, cytokine secretion in intact tissue is spatially restricted and efficiently captured by neighbouring cells^46^,^47^. Cells exposed to TNF *in vivo*, in a tissue or tumour, should therefore experience a “puff”, or pulse, of TNF at local concentrations that far exceed those measured in blood. Therefore a locally concentrated short TNF pulse, which we showed to be sufficient to initiate cell fate decisions, is also likely to be biologically relevant.

**Figure 6:**
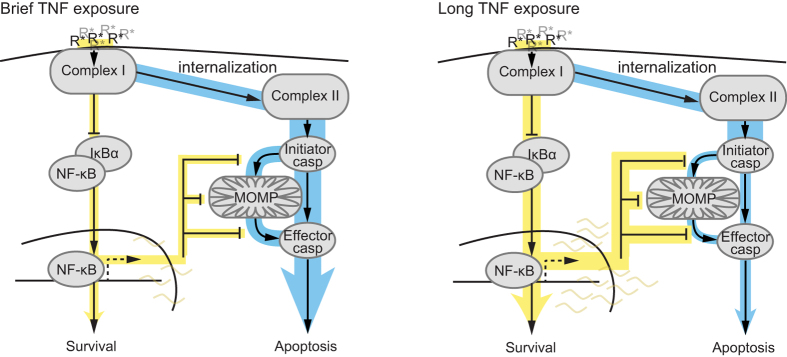
A working model for TNF duration-dependent flux through pro-survival and pro-apoptotic signalling pathways. Schematics of the flux through pro-survival NF-κB and pro-apoptotic caspase pathways under scenarios of brief (left) and long (right) high-concentration TNF exposures. In both scenarios, a large fraction of TNF receptors is ligand-bound (R*) and both Complex I and Complex II are efficiently formed. However, with brief TNF exposure NF-κB activation is not sustained and pro-survival gene expression is weak allowing stronger flux through the initiator caspases, mitochondrial outer membrane permeabilisation (MOMP) and effector caspases and promoting early apoptosis in some cells (left). By contrast a long TNF exposure allows sustained NF-κB activation reducing flux through the pro-apoptotic branch of the signalling network.

Based on the current understanding of TNF-induced signalling, cell-to-cell differences in the formation of Complex I and Complex II should alter the relative strength of pro-survival and pro-apoptotic signals. TNF dissociates slowly from TNFR1 (ref. 32 and Fig. 2d), over a timescale longer than that of internalization of TNF-bound receptor complexes^19^,^20^,^33^. Considering this slow dissociation, two aspects of our results are surprising: 1) that cell death timing differed following a short pulse vs. continuous treatment and 2) that fewer HeLa cells died after a 60-min pulse than after a shorter pulse or continuous treatment. Our working model for these observations is that the duration of TNF exposure may alter the relative strength of crosstalk between pro-survival and pro-apoptotic signals. While a high fraction-bound of TNFR1 is effectively reached during a short exposure to TNF, sufficient for formation of Complex II and caspase activation, the lack of continued receptor-ligand interactions following the pulse cannot sustain long-term Complex I and NF-κB activity. The short-pulse scenario therefore leads to weaker overall activation of the pro-survival pathway (Fig. 6, left). By contrast, a longer TNF exposure and the resulting sustained NF-κB activity could yield a more efficient activation of NF-κB-mediated pro-survival signals. These pro-survival signals could, in turn, block the slow time-scale pro-apoptotic caspase signals leading to an overall weaker flux through the pro-apoptotic pathway (Fig. 6, right). Under this working model, a pulse of a particular duration that optimizes activation of the pro-survival pathway relative to the pro-apoptotic pathway may therefore minimize cell death, akin to our observation of a local minimum of cell death after a 60-min pulse of TNF.

Nevertheless, our results do not exclude the possibility that TNF-treated cells may also integrate additional pathways downstream of Complex I and Complex II to arrive at a duration-specified cell fate decision. Specifically, TNF-induced autocrine signals are known to contribute to the fate decision^48^ and these secreted pro-survival and pro-apoptotic factors are likely diluted during flow and wash-out experiments. Analogously, the flow of blood and interstitial fluids could influence the extent and timing of TNF-induced cell death *in vivo*. The study of the interplay between intracellular and extracellular signals in the context of a TNF response will require experimental paradigms that integrate measurements and manipulations of both types of signals.

Overall, our results show that treatment duration is an important mediator of TNF-induced signalling and cell death decision and that in certain contexts reaching the highest fractional kill may not require maximizing the duration of exposure to a pro-death stimulus. These findings meet a growing body of work showing that signalling dynamics as well as the timing and sequence of drug addition can all influence cell fate decisions^36^,^49^,^50^,^51^,^52^,^53^,^54^. It will be interesting in the future to examine the effect of exposure duration, and the interplay with ligand-receptor-affinity, in other cellular signalling networks.

## Materials and Methods

### Cell culture and treatment of cells with TNF

HeLa cells (ATCC, VA) stably expressing EGFP-RelA (described in ref. 25) and HeLa stably expressing IC-RP (described in ref. 55) were cultured in Dulbecco’s Modified Eagle Medium (DMEM) supplemented with 10% FBS, 100 U/ml penicillin, 100 μg/ml streptomycin and 0.2 mM L-glutamine (Invitrogen, MA) at 37 °C and 5% CO_2_. Kym-1 cells^56^ were cultured in RPMI1640 supplemented with 10% FBS, 100 U/ml penicillin, 100 μg/ml streptomycin and 0.2 mM L-glutamine (Invitrogen, MA) at 37 °C and 5% CO_2_. For experiments in microfluidic devices, TNF treatments are described below. For experiments in 96-well imaging plates (BD Biosciences, CA), on day 1, HeLa cells were seeded at ~4000 cells/well, Kym-1 cells were seeded at ~6000 cells/well. On day 2, culture medium was replaced with medium with 200 U/ml IFNγ (Roche, IN) to sensitize cells to TNF-induced apoptosis (it is necessary for HeLa cells which are not sensitive otherwise, but here we used it as well for Kym-1 for consistency). IFNγ is, like TNF, an inflammatory cytokine and it sensitizes many cancer cell lines to TNF-induced apoptosis, partly via increased initiator caspase-8 expression^35^,^36^,^37^,^38^,^39^. On day 3, two hours prior to TNF treatment, culture medium was replaced (with IFNγ) and 24 hrs after IFNγ treatment, complete medium with or without TNF was spiked into wells to yield the indicated final TNF concentrations. After the specified duration, TNF-containing medium was removed, cells were rapidly washed three times and then incubated in the appropriate medium without TNF (with or without IFNγ) for the duration of the experiment. To control for any effects of washes and medium exchange, these were also performed for cells treated continuously (medium replaced to medium containing the appropriate concentration of TNF, with or without IFNγ at the start of treatment). Care was taken to use only conditioned and warmed medium for all the washes and media changes during the experiment, to minimize disturbances to the cells.

### Device fabrication and operation

Microfluidic chips with two inlets and three chambers for cell culture (illustrated in Fig. 1a) were made in PDMS (Sylgard 184, Dow Corning Corporation, MI) and bonded to a No. 1.5 glass coverslip (ThermoScientific, MA). Pipet tips (200 μl) were inserted into 1/16″ tygon tubing connected to steel tubes (16-gauge) into punched inlets and outlets to act as fluid reservoirs.

To prepare for experiments, devices were sterilized with 70% ethanol then abundantly flushed with sterile PBS. Channel surfaces were pacified by flushing the devices with complete culture medium and incubating >12 hrs. Cells were then seeded at appropriate density and allowed to adhere for 12 hrs under no-flow conditions (all reservoirs at equal height). Medium was replaced then and again 24 hrs and 4 hrs before an experiment.

For treatment with a pulse of TNF, medium with TNF at the final desired concentration was prepared with 1 μg/ml Alexa Fluor^®^ 647 conjugated-BSA (Life Technologies, MA). The device was securely mounted on a custom stage for a BD Pathway 855 BioImager and flow allowed to reach a steady state in the pre-pulse mode (Fig. 1a) while monitoring by imaging the Alexa-647-BSA epifluorescence signal. To pulse cells with TNF, the ‘medium + TNF’ reservoir was temporarily raised manually (Fig. 1a). Pulse duration was verified by imaging the Alexa-647-BSA epifluorescence signal.

### Live-cell imaging and analysis

For all live-cell experiments, HeLa cells stably expressing EGFP-RelA or IC-RP were imaged for 30 min before addition of TNF. Wide-field epifluorescence and transmitted light live-cell imaging was done in an environmentally controlled chamber (37 °C, 5% CO_2_) on the BD Pathway 855 BioImager (BD Biosciences, CA) using a UAPO/340 20x objective (0.75 NA; Olympus, MA), capturing images at 3 min intervals for EGFP-RelA HeLa and at 10 min intervals for IC-RP HeLa. Data was extracted from flat-field and background corrected time-lapse images using ImageJ. Mean fluorescence intensity (MFI) of nuclear EGFP-RelA was collected for each cell at each time point using custom scripts. For caspase activity measurements, the ratios of MFI in the CFP and YFP channels were calculated. For Kym-1 cells, the time of cell death was determined by finding for each cell the first frame where membrane blebbing, a hallmark of apoptosis, was evident. For all experiments, cells that overlapped, left the imaging field or divided within an hour of TNF treatment were excluded from analysis.

### Determination of a nuclear EGFP-RelA fold-change threshold for cellular response to TNF

To estimate the error in determining whether a cell has had a transcriptionally significant response to TNF based on the fold-change of nuclear NF-κB in a cell (Fig. 2a), we considered two parameters. The first parameter is the ‘cut-off value for transcript number’ (*transcript*_*cutoff*_) which we defined as the minimum number of transcripts expected from a cell that has undergone a transcriptional response to TNF. We thus assumed that a cell with *transcript*_*cell*_ ≥ *transcript*_*cutoff*_ would have been likely to be transcriptionally activated in response to a stimulus. The second parameter is the fold-change threshold (*FC*_*thresh*_), a parameter that defines the value of ‘nuclear fold change’ at which significant transcriptional activation should occur. Because our dataset contains a pool of paired values that describe the maximum fold change (*FC*_*cell*_) as well as the transcript number (*transcript*_*cell*_) for each of hundreds of single cells exposed to a range of TNF concentrations, we evaluated the total error as follows:

1. Assume that a given value of *transcript*_*cutoff*_ correctly partitions the dataset into transcriptionally responsive and non-responsive cells. From this it follows that we assumed that a cell with *transcript*_*cell*_ ≥ *transcript*_*cutoff*_ mRNAs has undergone a transcriptional response, whereas a cell with fewer transcripts (i.e. *transcript*_*cell*_ < *transcript*_*cutoff*_) has not.
2. For each value of *FC*_*thresh*_, quantify the number of false positives and false negatives. False positives were defined as a cell for which *FC*_*cell*_ ≥ *FC*_*thresh*_, but *transcript*_*cell*_ < *transcript*_*cutoff*_. Thus this cell had a greater than threshold response, as indicated by change in nuclear NF-κB, but was not transcriptionally activated based on assumption in (1). False negatives were defined as a cell for which *FC*_*cell*_ < *FC*_*thresh*_, and *transcript*_*cell*_ ≥ *transcript*_*cutoff*_. Thus this cell had a sub-threshold response based on change of nuclear NF-κB, yet was transcriptionally activated as per (1).
3. The total error for a value of *FC*_*thresh*_ is the total number of false positives and false negatives divided by the total number of cells. The total error was calculated across a range of *FC*_*thresh*_ values in increments of 0.01.

Finally, the nuclear EGFP-RelA fold-change threshold for cellular response to TNF was defined as the fold-change threshold (*FC*_*thresh*_) where total error is minimized.

### Simulations of liquid flow in the devices

Three-dimensional simulations were carried out using COMSOL Multiphysics 3.5 (COMSOL, Inc., MA), modelling the actual shape and dimensions of the device. Simulations solved Navier–Stokes equations with the different boundaries conditions and the following assumptions: 1) fluids similar to water (incompressible Newtonian fluid with a density of 998.2 kg.m^−3^, and a dynamic viscosity of 0.001 N · s m^−2^), 2) no-slip boundary conditions on the channel walls and 3) steady-state conditions were reached. The hydrostatic pressures at each inlets were calculated from its height relative that of the outlet reservoir.

### Model of bimolecular surface reaction

Binding of TNF to TNFR1 and TNFR2 was modelled as independent reversible receptor-ligand interactions with constant ligand concentration^31^:


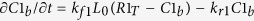



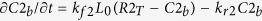


*C*1_*b*_ and *C*2_*b*_ are the concentrations of TNF-bound TNFR1 and TNFR2, respectively, *L*_0_ is the concentrations of TNF, *R*1_*T*_ and *R*2_*T*_ are the total concentrations of receptors (assuming 3,000 TNFR1 molecules per cell for HeLa and Kym-1, and 30,000 TNFR2 molecules per cell for Kym-1), *k*_*f*1_ and *k*_*r*1_ are the association (1.833 × 10^7^ M^−1^s^−1^) and dissociation rate (3.5 × 10^−4^ s^−1^) constants for TNF-TNFR1 and *k*_*f*2_ and *k*_*r*2_ are the association (2.5 × 10^7^ M^−1^s^−1^) and dissociation rate (0.011 s^−1^) constants for TNF-TNFR2 (as reported in a previous study^32^). We surmise that constant ligand concentration is an appropriate approximation as with a very high medium:cell volume ratio, the number of ligand molecules vastly surpasses that of receptors. Simulations were carried out in MatLab (MathWorks, MA) using the ode45s solver.

## Additional Information

**How to cite this article**: Lee, R. E. C. *et al*. NF-κB signalling and cell fate decisions in response to a short pulse of tumour necrosis factor. *Sci. Rep.* **6**, 39519; doi: 10.1038/srep39519 (2016).

**Publisher's note:** Springer Nature remains neutral with regard to jurisdictional claims in published maps and institutional affiliations.

## Supplementary Material

Supplementary Movie S1

Supplementary Movie S2

Supplementary Movie S3

Supplementary Information
