# Supplementary material for: NF-κB signalling and cell fate decisions in response to a short pulse of tumour necrosis factor
Source: Sci Rep. 2016 Dec 22;6:39519. doi: 10.1038/srep39519 (PMC5177917; doi:10.1038/srep39519)
Supplement: Supplementary Information [file srep39519-s4.pdf]

NF-κB signaling and cell fate decisions in response to a short pulse of tumor necrosis factor

Authors: Robin E. C. Lee[1,2,6,†], Mohammad A. Qasaimeh[1,2,3,7,†], Xianfang Xia[1,2], David Juncker[3,4,5] and Suzanne Gaudet[1,2,*]

Supplementary Information

Legends for Supplementary Movies……………………………………………………………2

Supplementary Figures S1-S8…………………………………………………………3-10

Supplementary Movie S1. A pulse of stimulus in the Y-channel device. Time-lapse epifluorescence imaging of the laminar streams ("Medium + Alexa647-conjugated BSA" is red; "Medium" is dark) in the device chambers and channel during a 10-sec pulse. Images were streamed continuously, and are shown at 2X real time.

Supplementary Movie S2. Nuclear translocation of EGFP-RelA in HeLa cells treated with a brief pulse of TNF. EGFP-RelA nuclear translocation as monitored by time-lapse epifluorescence imaging of EGFP-RelA HeLa cells before and after treatment with a 30-sec pulse of 100 ng/ml TNF. Images were taken at 3-min intervals.

Supplementary Movie S3. Apoptotic cell death of HeLa cells in response to TNF treatment. ECFP/EYFP ratio as monitored by time-lapse epifluorescence imaging of IC-RP HeLa cells during continuous treatment with 100 ng/ml TNF following a 24-hr pre-treatment with 200 U/ml IFNγ. The ECFP/EYFP ratio is represented in grayscale (black is low, white is maximal), images were taken at 10 min intervals.

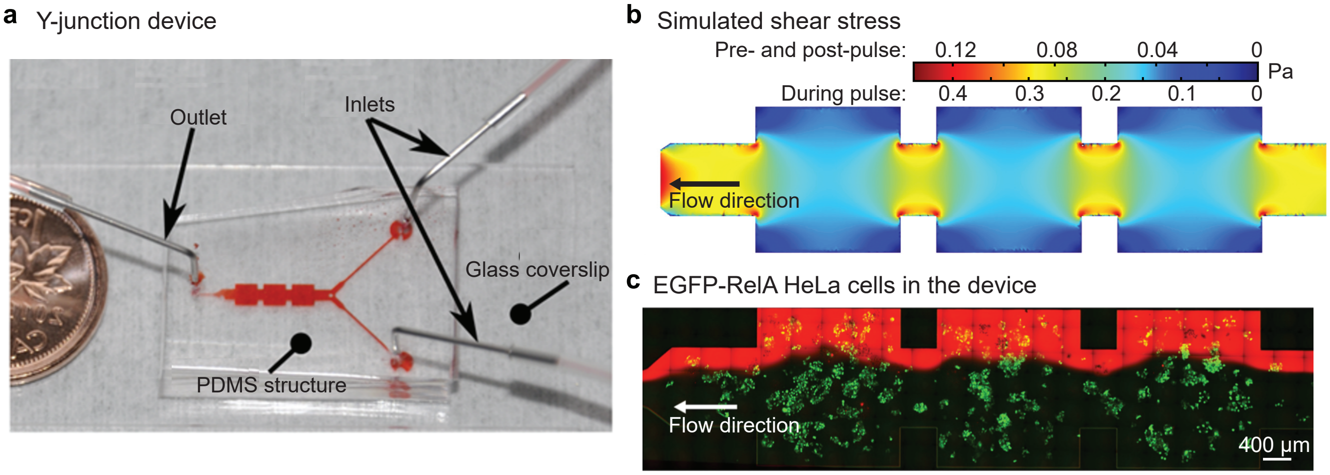

Supplementary Figure S1. Microfluidic system to stimulate cells with a pulse of TNF under minimal shear stress. (**a**) Image of the Y-junction microfluidic device, filled with a red dye. (**b**) Heat map of the simulated shear stress in a model of flow in the microfluidic system. Scale bar indicates the color correspondence for forces predicted to occur pre- and post-pulse (above) and during pulse (below). (**c**) Montage of tiled images showing the cell growth chambers and connecting channel. Images of the EGFP-RelA HeLa cells (green) and Alexa647-BSA (red) were overlaid.

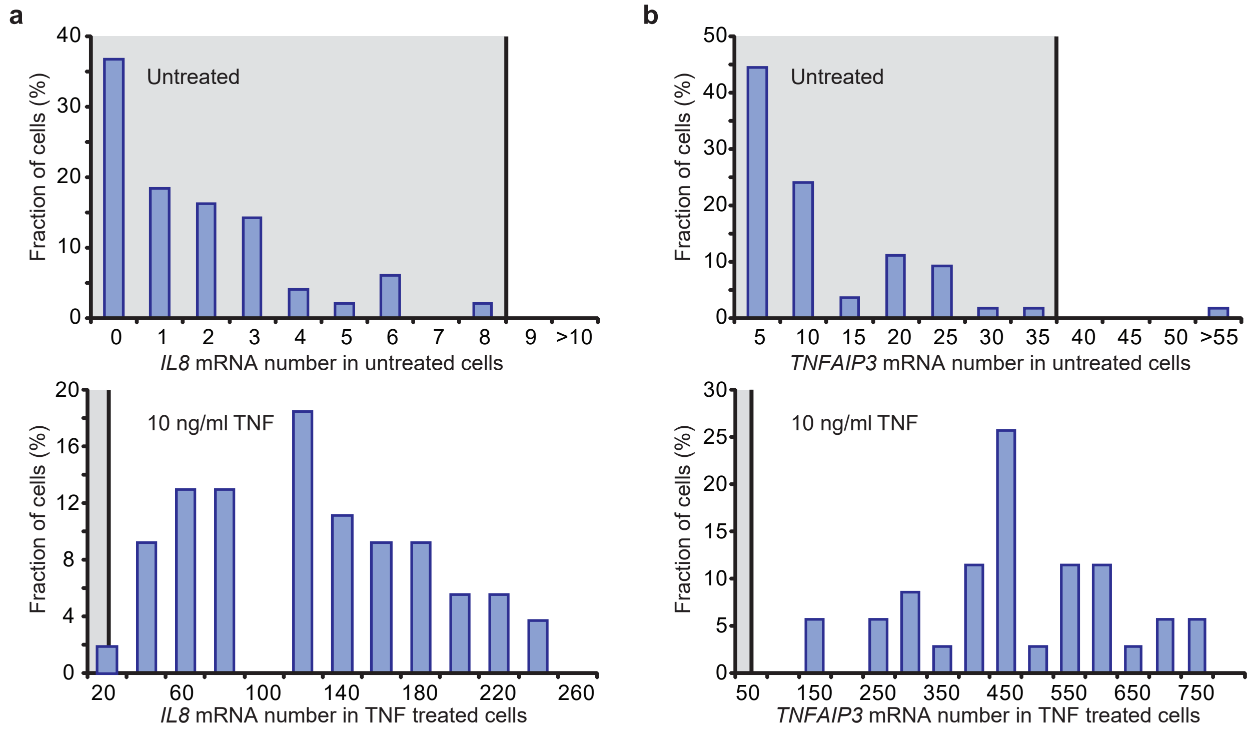

Supplementary Figure S2. Same-cell nuclear EGFP-RelA translocation and transcript number data allow classification of cells as TNF treatment 'responders' or 'non-responders'. (**a&b**) Histograms of the transcript number distributions in untreated (top) and TNF-treated (10 ng/ml; bottom) EGFP-RelA HeLa cells for *IL8* (a) and *TNFAIP3* (b). Data re-analysed from Lee et al. (2014). The light gray zones indicate a threshold for the 'baseline' number of transcripts expected to be observed in untreated (or 'non-responsive') HeLa cells (< 8 for *IL8* and < 35 for *TNFAIP3*). Distributions were generated from data collected on n = 50 to 156 cells from at least two independent experiments.

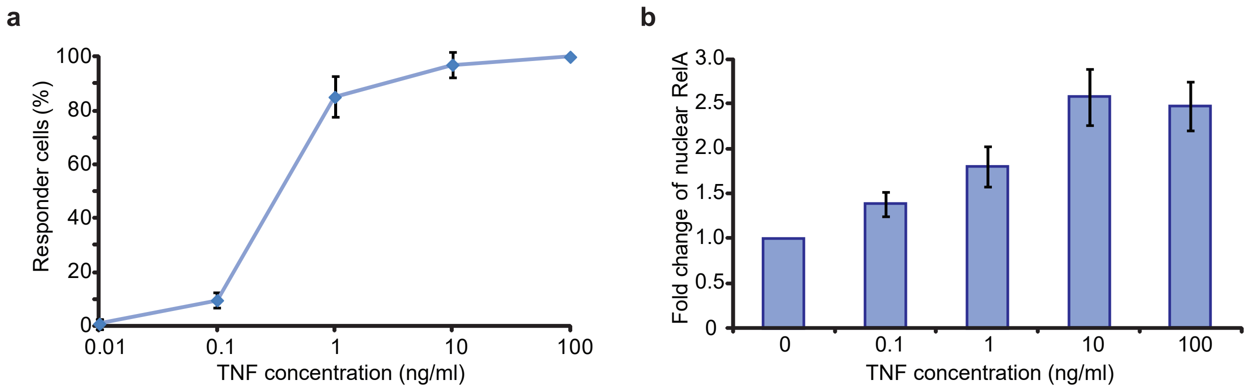

Supplementary Figure S3. Fraction of responding cells and their average nuclear FP-RelA fold change are TNF dose-dependent. (**a**) Dose-response curve of the EGFP-RelA HeLa cells treated with TNF, as expressed as the percentage of 'responder' cells (with nEGFP-RelA fold change > 1.22) observed at each TNF concentration. (**b**) Bar graph of the average fold change in nuclear EGFP-RelA in 'responder' cells observed when treating EGFP-RelA Hela cells continuously with the indicated TNF concentrations. Error bars represent the standard error of the mean (S.E.M.). For both panels, we re-analyzed a dataset orginally published in (Lee *et al.*, 2014, Ref. 25); this dataset combined data from three to six independent experiments, resulting in between 78 and 198 total cells per condition.

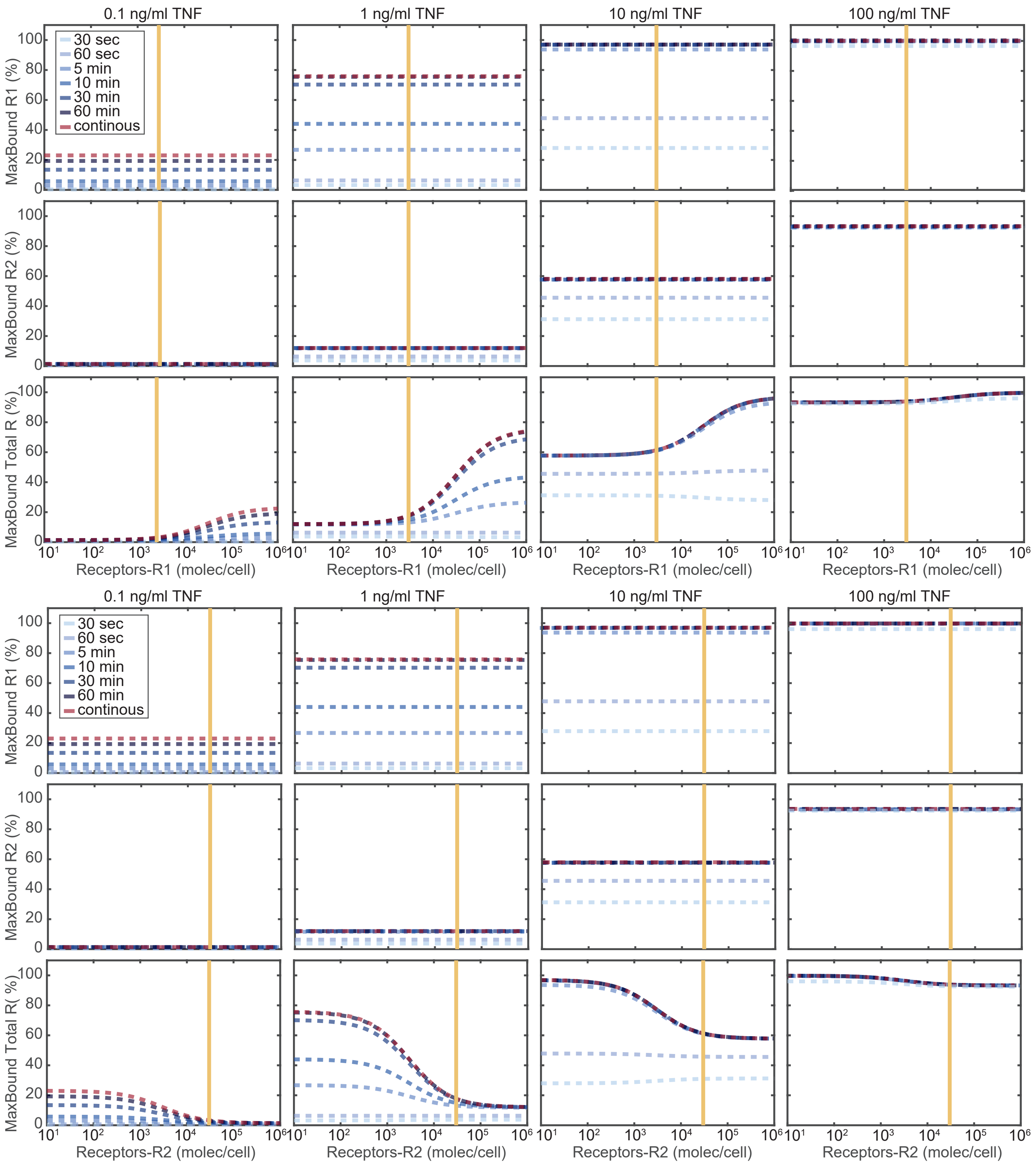

Supplementary Figure S4. The abundance of TNFR1 and TNFR2 shows little impact on simulated bound receptor fraction. Plots show the simulated relationship between initial TNFR1 (top panel) or TNFR2 (bottom panel) concentration and maximal bound TNFR1, TNFR2 and total receptors (top, middle and bottom rows respectively) following TNF stimulation of the indicated durations. Receptor concentration was uniformly sampled in the exponent for values between 10^2 to 10^7 proteins per cell; all other parameters were set at their default value. Vertical bars represent the measured concentrations for TNFR1 (3,000 molec/cell for HeLa and Kym-1) and TNFR2 (30,000 molec/cell for Kym-1) (Grell *et al*., 1998; Ref. 32). Maximal bound total receptors varies because as TNFR1 or TNFR2 abundance is varied, the ratio or TNFR1:TNFR2 varies.

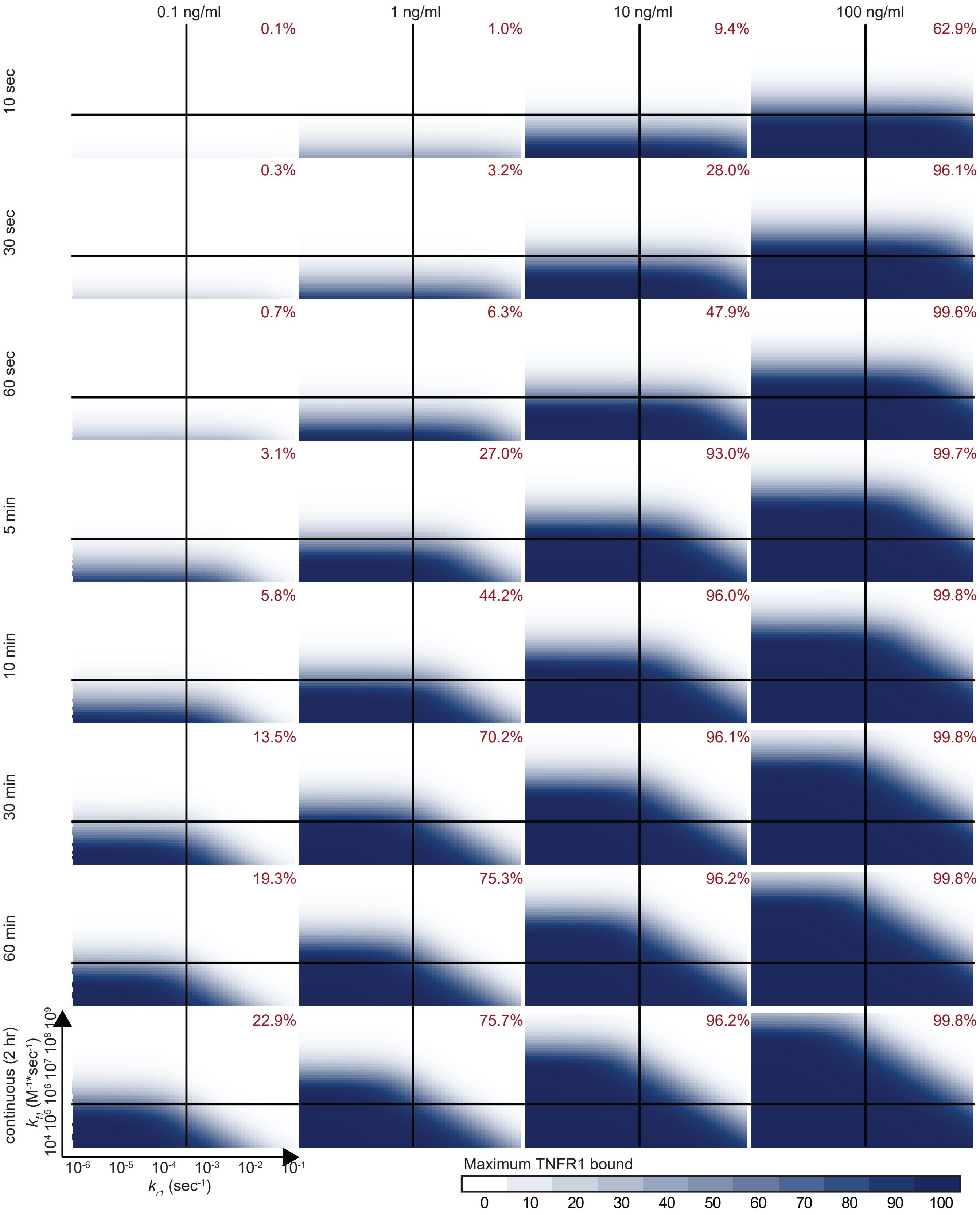

Supplementary Figure S5. Binding and unbinding rates of TNF to TNFR1 non-linearly impact the maximal fraction of TNFR1 bound. Heatmaps of the maximum fraction (%) of TNFR1 bound to TNF in 2-hr simulations where k_{r1} and k_{f1} were varied linearly over the exponent. The fraction bound is also dependent on duration of the TNF pulse (rows) and TNF concentration (columns). Vertical black lines indicate the default k_{r1} value (Grell *et al.*, 1998; Ref. 32); horizontal black lines indicate the default k_{f1} value (Grell *et al.*, 1998; Ref. 32); dark red numbers are the values for fraction of TNFR1 bound at their intersection.

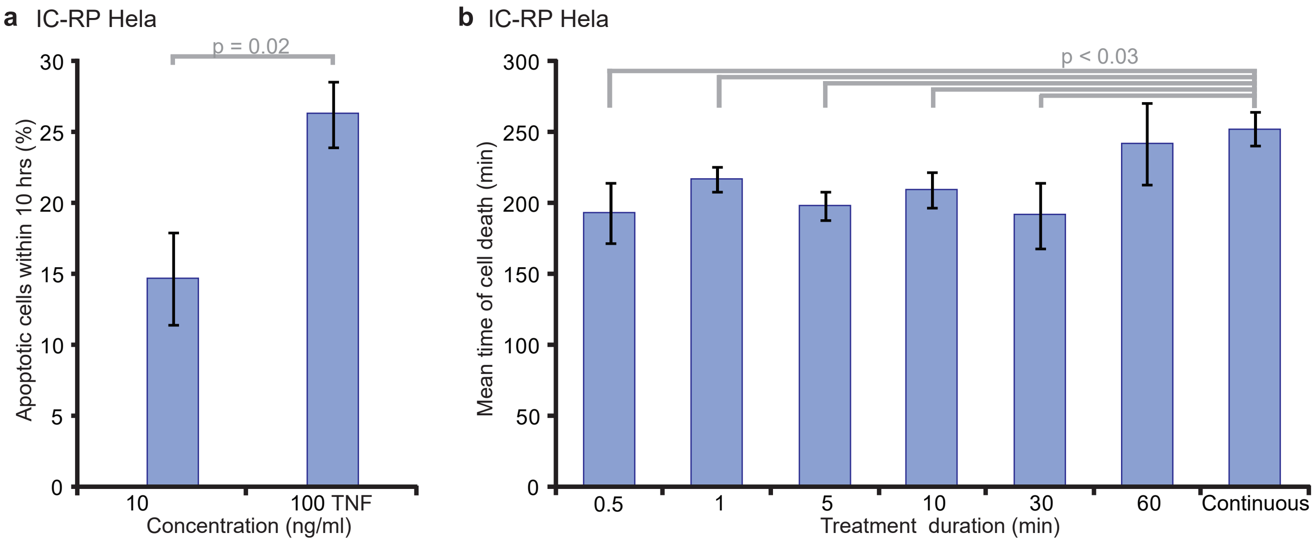

Supplementary Figure S6. Apoptosis timing shows subtle but statistically significant differences when comparing a TNF pulse to continuous treatment in IFNγ-pre-treated cells. (**a**) Bar graph of the percentages of apoptotic cells in IC-RP HeLa cells continuously treated with the indicated TNF concentration for 10 hours. Error bars represent the standard deviation from three biological replicates; p-value for an unpaired two-tailed t-test, n = 67 to 100 cells per replicate per condition. (**b**) Bar graph of the mean cell death time of IC-RP HeLa cells treated with 100 ng/mL TNF for the indicated duration. Error bars represent the standard error of the mean from n = 5 independent biological replicated experiments. P-value shows significantly shorter mean cell death time as determined by a paired one-tailed t-test.

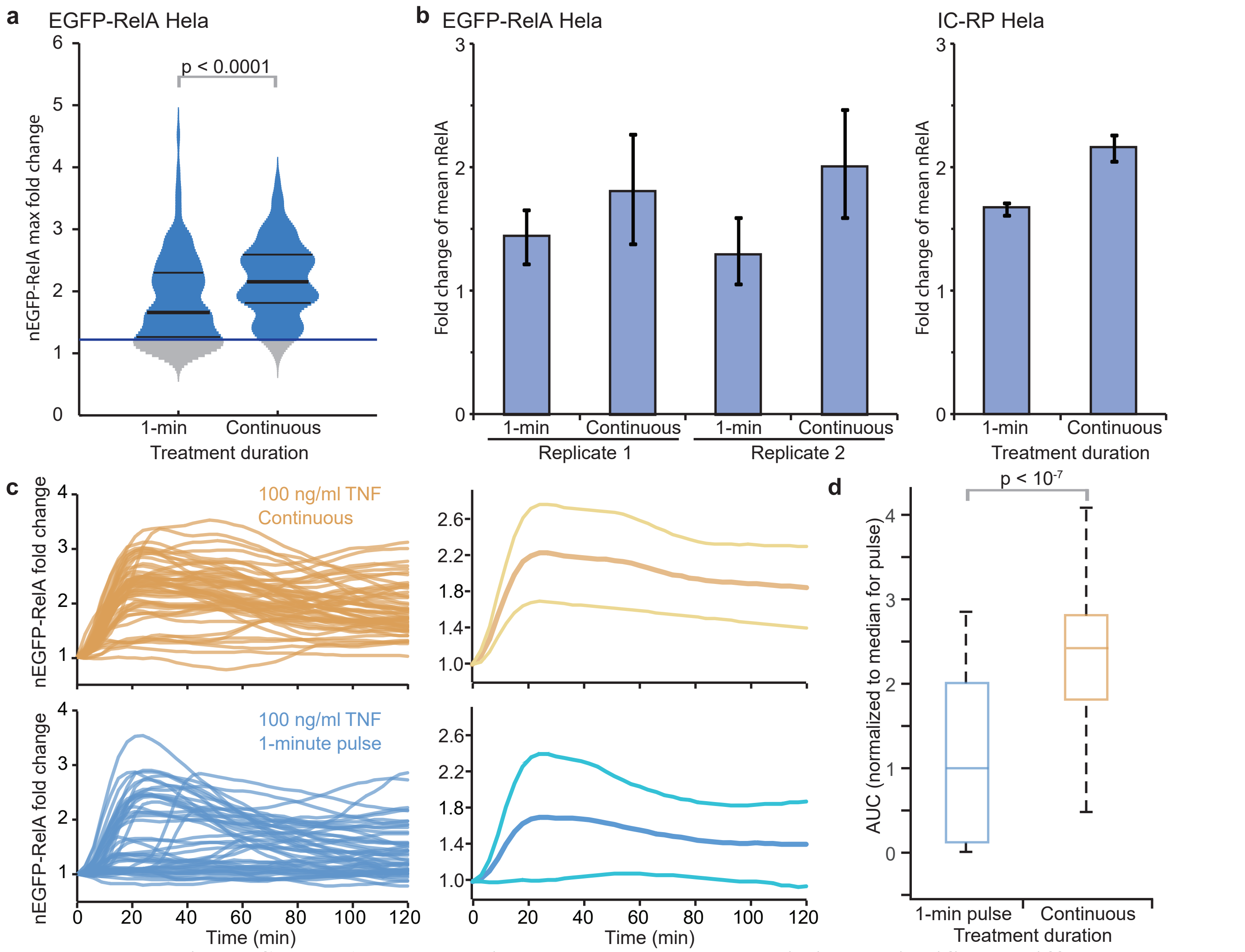

Supplementary Figure S7. RelA translocation shows subtle but statistically significant differences when comparing a 1-min TNF pulse to continuous treatment in IFNγ-pre-treated cells. (**a**) Violin plots of the relative frequency distributions of maximum fold change in nuclear EGFP-RelA (nEGFP-RelA) in EGFP-RelA treated with 100 ng/ml TNF for the indicated duration and imaged for 60 min by live-cell microscopy. Above the 1.22 fold change threshold (blue line) cells were classified as 'responders' (blue), and below as 'non-responders' (gray). The median (thick black line) and upper and lower quartiles (thin black lines) are marked. P-value of a Kolmogorov-Smirnov test of the equality of the distributions is reported. Data from two independent experiments were combined, n = 158 cells for 1-min pulse and n = 99 cells for continuous and violin plots were generated in MatLab from smoothened histograms with normal kernel. (**b**) Bar graphs of the fold change of the mean nuclear RelA (mean nRelA at t = 60 min divided by mean nRelA at t = 0 min) observed by live-cell imaging in EFGP-RelA HeLa (left) and by immunofluorescence using a mouse monoclonal antibody against RelA (Santa Cruz Biotechnologies) in IC-RP HeLa (right). Error bars represent the 95% confidence interval determined by bootstrapping; n = 84 and 48 cells for replicate 1, n = 74 and 51 cells for replicate 2 for EGFP-RelA HeLa and n = 4996 cells (t = 0 min), n = 2161 cells (t = 60 min, 1-min pulse) and n = 1183 cells (t = 60 min, continuous treatment) for IC-RP HeLa. (**c**) Single-cell nuclear EGFP-RelA time courses after continuous treatment (orange, n = 31 cells) or 1-min pulse (blue, n = 30 cells) of TNF in cells pretreated with IFNγ (left). Average (thick line) and standard deviations (thin lines) of time courses plotted on the right. (**d**) Box plots of the distributions of the area under the curve (AUC) for trajectories in (c) showing median, top and bottom quartiles (box) and 5th and 95th percentiles (whiskers). P-value indicates a significant difference between continuous- and pulse-treated cells (one-tailed t-test).

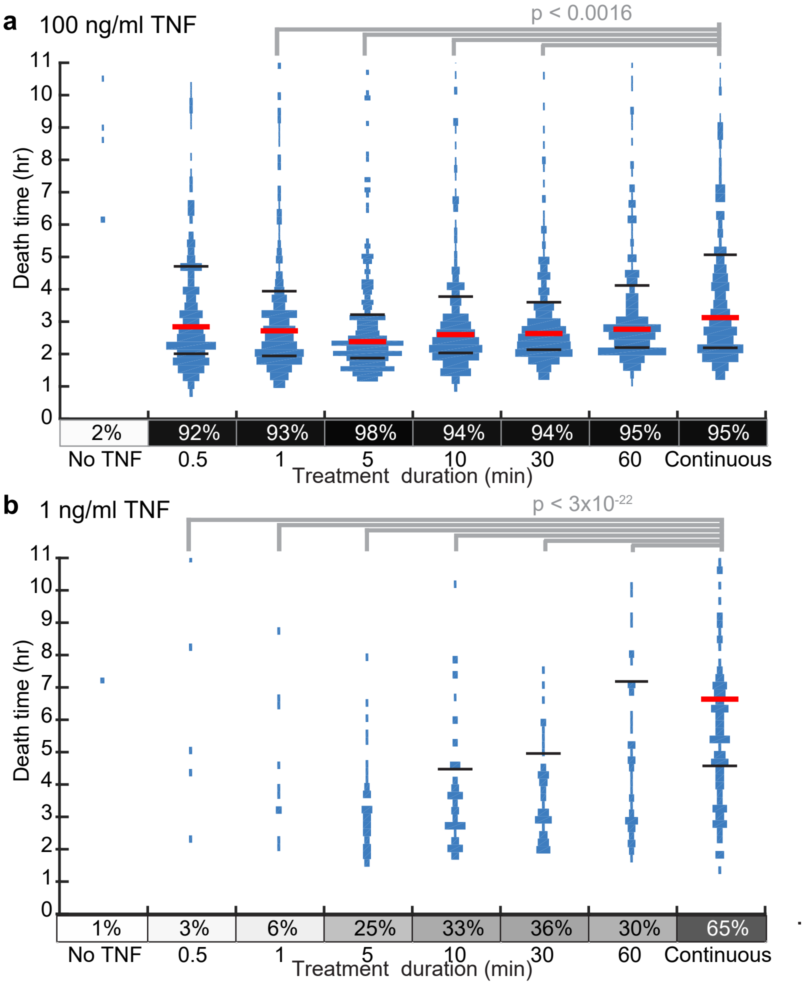

Supplementary Figure S8. Cell death occurs on average earlier in Kym-1 treated with a short TNF pulse than in those treated continuously. (**a** & **b**) Violin plots of the relative frequency distributions of cell death times for Kym-1 cells treated with 100 ng/ml (a) or 1 ng/ml (b) TNF for the indicated duration after a 24-hr pre-treatment with 200 U/ml IFNγ. The median (red line) and upper and lower quartiles (black lines) are marked and average percentages of dead cells at t = 10 hr are indicated in heat maps below the graphs. To obtain median and quartile values, surviving cells were assigned a death time of 25 hr and therefore lower quartile, median and upper quartile are indicated only if there is more than 25%, 50% or 75% cell death, respectively. Kolmogorov-Smirnov tests of the equality of the distributions show that cell death time distributions are different from that obtained with continuous treatment at the same concentration, except for the 30-sec pulse treatment. Data from four (100 ng/ml TNF; panel a) or three (1 ng/ml TNF; panel b) independent experiments were combined for a total of between 131 and 278 cells per condition; violin plots were generated in MatLab from histograms without smoothing, bin size of 7.5 min.
